# Supplementary material for: The Role of Self-as-Context as a Self-Based Process of Change in Cancer-Related Pain: Insights from a Network Analysis
Source: Healthcare (Basel). 2025 Oct 28;13(21):2722. doi: 10.3390/healthcare13212722 (PMC12607370; doi:10.3390/healthcare13212722)
Supplement: Supplementary file 1 [file healthcare-13-02722-s001.zip › healthcare-3700148-supplementary.pdf]

Supplementary Material (SM)

Supplementary Material S1: Self-as-Context Scale (SACS) Original (EN) and Greek Adaptation (GR)

**SACS A/A**	**SACS Items**
**1-C**	▪ When I am upset, I am able to find a place of calm within myself. (EN)<br>▪ *Όταν είμαι αναστατωμένος/-η, είμαι ικανός/-ή να βρω ένα ήσυχο μέρος μέσα μου. (GR)*
**2-C**	▪ I have a perspective on life that allows me to deal with life's disappointments without getting overwhelmed with them. (EN)<br>▪ *Έχω μια προοπτική στη ζωή που μου επιτρέπει να αντιμετωπίζω τις απογοητεύσεις της ζωής χωρίς αυτές να με κυριεύουν. (GR)*
**3-T**	▪ Despite the many changes in my life, there is a basic part of who I am that remains unchanged. (EN)<br>▪ *Παρά τις πολλές αλλαγές στη ζωή μου, υπάρχει ένα βασικό μέρος του ποιος/-α είμαι που παραμένει αμετάβλητο. (GR)*
**4-T**	▪ As I look back upon my life so far, I have a sense that part of me has been there for all of it. (EN)<br>▪ *Καθώς ανατρέχω στη ζωή μου μέχρι τώρα, έχω την αίσθηση ότι μέρος του εαυτού μου ήταν εκεί για όλα αυτά. (GR)*
**5-C**	▪ I allow my emotions to come and go without struggling with them. (EN)<br>▪ *Αφήνω τα συναισθήματά μου να έρχονται και να φεύγουν χωρίς να παλεύω μ' αυτά. (GR)*
**6-C**	▪ I am able to notice my changing thoughts without getting caught up in them. (EN)<br>▪ *Είμαι ικανός/-ή να παρατηρήσω τις μεταβαλλόμενες σκέψεις μου χωρίς να αναλώνομαι σ' αυτές. (GR)*
**7-T**	▪ There is a basic sense I have of myself that doesn't change even though my thoughts and feelings do. (EN)<br>▪ *Υπάρχει μια βασική αίσθηση που έχω για τον εαυτό μου ότι δεν αλλάζει παρόλο που οι σκέψεις και τα συναισθήματά μου αλλάζουν. (GR)*
**8***	▪ Though I have had many roles in life, I have always had a sense of myself that is stable and enduring. (EN)<br>▪ *Αν και είχα πολλούς ρόλους στη ζωή μου, είχα πάντα μια αίσθηση του εαυτού μου σταθερή και ανθεκτική. (GR)*
**9-T**	▪ Even though there have been many changes in my life, I'm aware of a part of me that has witnessed it all. (EN)<br>▪ *Παρόλο που έχουν υπάρξει πολλές αλλαγές στη ζωή μου, αναγνωρίζω πως ένα μέρος του εαυτού μου τα έχει δει όλα. (GR)*
**10-T**	▪ I am able to access a perspective from which I can notice my thoughts, feelings, and emotions. (EN)<br>▪ *Είμαι ικανός/-ή να έχω πρόσβαση σε μια προοπτική μέσα από την οποία μπορώ να παρατηρώ τις σκέψεις και τα συναισθήματά μου. (GR)*
**11-T**	▪ When I think back to when I was younger, I recognize that a part of me that was there then is still here now. (EN)<br>▪ *Όταν σκέφτομαι τότε που ήμουν νεότερος/-η, αναγνωρίζω ότι ένα μέρος του εαυτού μου που ήταν εκεί τότε είναι ακόμη εδώ τώρα. (GR)*

Note-1: C: Centering items (referring to calm reactions to unwanted psychological experiences);
T: Transcending items referring to an invariant perspective-taking of what is characterized "observing self" (Zettle et al., 2018); SACS uses a 7-point Likert scale (e.g., 1 = completely disagree to 7 = completely agree) and higher scores indicate a greater sense of self-as-context. For the scoring of the centering subscale, items 1, 2, 5, 6 sum up and for the scoring of transcending subscale items 3, 4, 7, 9, 10, 11 sum up. The scale does not use reverse-scored items. (Zettle et al., 2018)
Note-2: Item 8 was not retained in the initial validation study (Zettle et al., 2018).

Supplementary Material S2: Network Edge Stability with Bootstrapped confidence Intervals of all Edges

Network

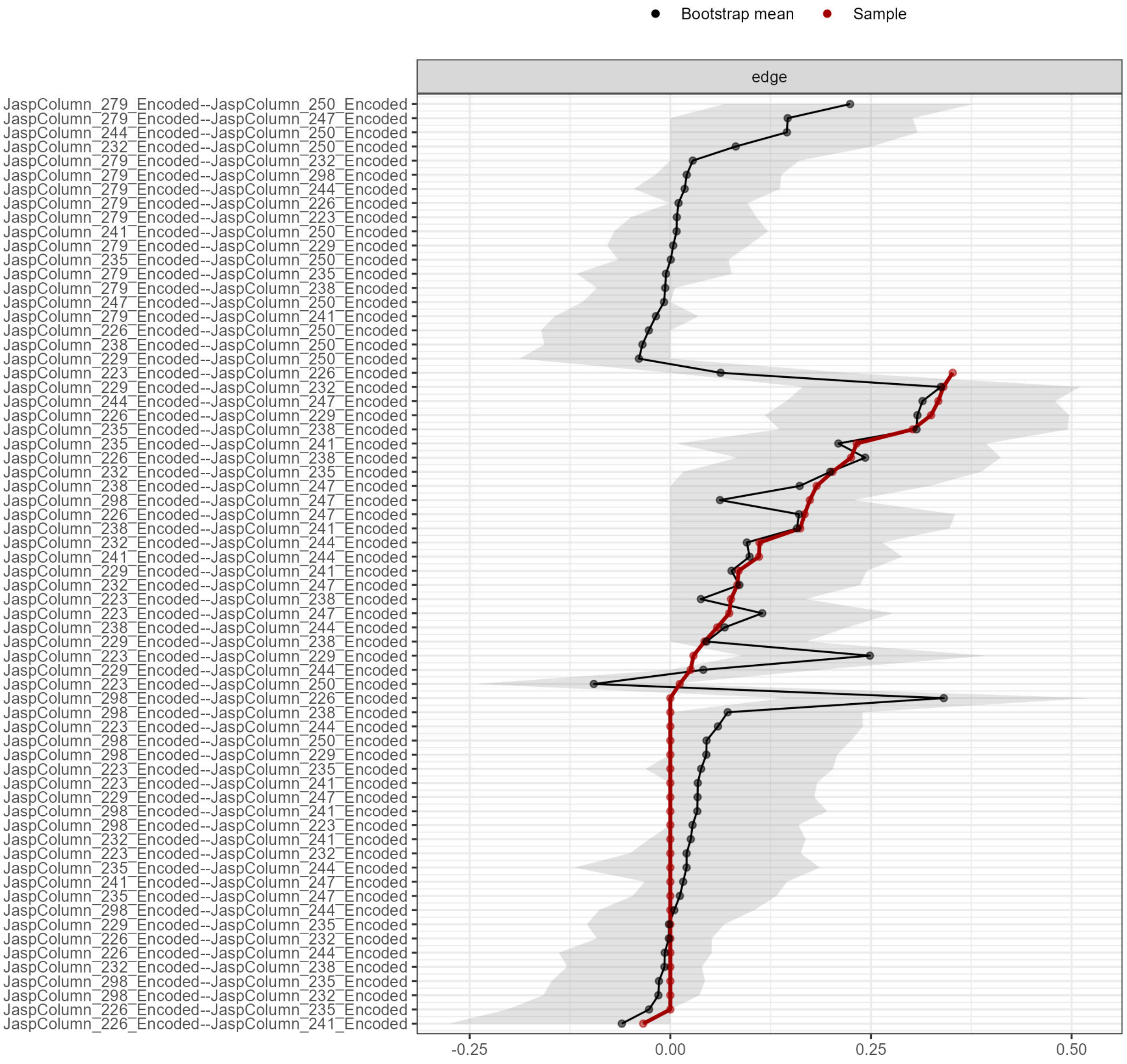

Supplementary Material S3: Network's Centrality Stability Coefficient Intervals.

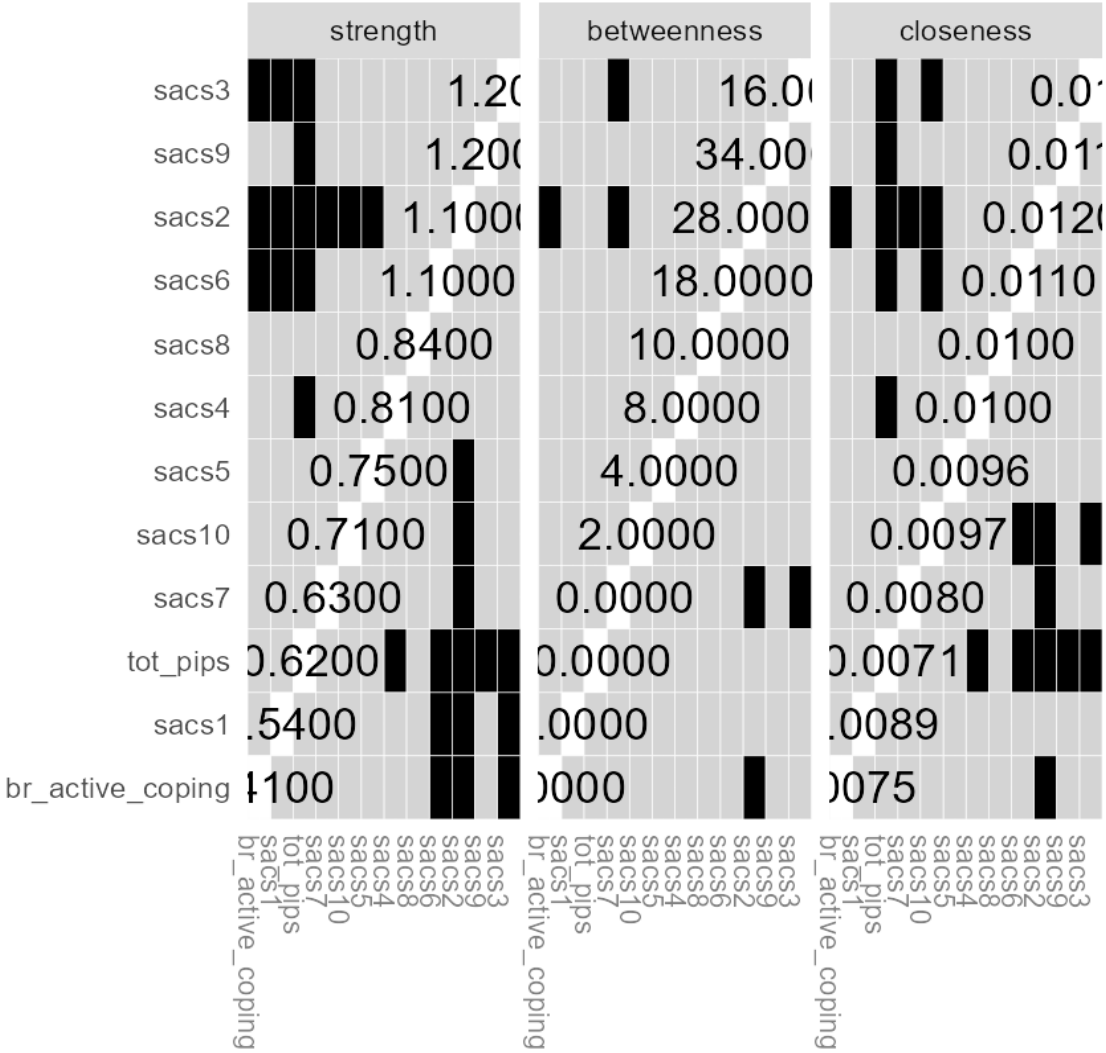

Note: The mean predictability of the overall network for strength was 0.825.

Supplementary Material S4: List of supporting organizations which assisted with the sample's recruitment

The following list includes the participating organisations:

- Association of Cancer Patients – Volunteers - Friends and Doctors of Athens,
- Cyclades Cancer Patients & Friends Association,
- Evros Cancer and Rare Diseases Association,
- Cancer Support Association of Rhodes,
- Association of Cancer Patients and Friends "Dynami Psychis",
- Association of Cancer Patients - Vulnerable Groups – Volunteers - Friends and Doctors of Preveza,
- AgaliaZo - Group of Volunteers Against Cancer of Western Greece,
- Association of Cancer Patients - Volunteers and Friends of Arta,
- Association of Cancer Patients of Giannitsa,
- Association of Women with Breast Cancer - Alma Zois Thessalonikis,
- Association of Cancer Patients and Friends of Argolida,
- Association of Cancer Patients of Veroia,
- Association of Cancer Patients of Larissa,
- Association of Women with Breast Cancer - Alma Zois Athens,
- Association of Cancer Patients of Edessa,
- Association of Women with Breast Cancer - Alma Zois Patras,
- FairLife L.C.C.,
- Association of Cancer Patients of Kozani,
- BeStrong and Volunteer Association of Evrytania Against Neoplastic Diseases
